# Colligative Property of ATP: Implications for Enteric Purinergic Neuromuscular Neurotransmission

**DOI:** 10.3389/fphys.2016.00500

**Published:** 2016-10-28

**Authors:** Arun Chaudhury, Vijaya S. R. Dendi, Wasique Mirza

**Affiliations:** ^1^GIM FoundationLittle Rock, AR, USA; ^2^Christus Trinity Mother Frances HospitalTyler, TX, USA; ^3^The Wright Center for Graduate Medical Education, The Commonwealth Medical CollegeScranton, PA, USA

**Keywords:** SLC17A9, VNUT, neurotransmission, transporter, enteric nervous system

## ATP, a common constituent of vesicles, reduces intravesicular osmotic pressure by polymerizing vesicular contents and reducing the number of individual free particles

A recent report by Estévez-Herrera et al. (2016) suggested that ATP, the energy coin, also a neurotransmitter, controls the osmotic pressure of vesicular contents (Estévez-Herrera et al., 2016). Since the discovery and description of quantal transmission (Katz, 1971; Bennett and Kearns, 2000), membrane-delimited packeted structures, the vesicles, have been morphologically identified and correlated with secretion in several secretory tissues, including nerve terminals of central and peripheral neurons, chromaffin cells, platelets, and insulin-secreting beta cells of the pancreas (Goyal and Chaudhury, 2013; Südhof, 2013; Thorn et al., 2016). Though it is now known for some time that ATP is concentrated in the vesicles, the demonstration that ATP, by virtue of being negatively charged, and its ability to associate with positively charged molecules like amines, agglomerates vesicular particles, is a significant finding. Vesicles do not release their contents randomly within the cytosol, but rather are transported to the membranes for exocytosis (Jena, 2009). An important contributor to this site-specific release of vesicular contents may be the constant fine tuning and maintenance of osmotic pressure isotonic with the cytosol of the vesicle-containing structure. In the light of such plausible dynamic regulation of osmotic pressure of vesicles, the demonstration of the likely role of ATP in regulating this vesicular osmotic pressure acquires importance (Estévez-Herrera et al., 2016). In this perspective, we discuss the implications of these findings on enteric purinergic inhibitory musculomotor neurotransmission.

## Vesicle membrane integrity is maintained by lowering intravesicular osmotic pressure

Osmotic property is a colligative property: it depends on the number of particles, as suggested by Raoult:

pV = nRT…standard gas equation
πV = nRT….π = osmotic pressure
π = [n/V]RT…note the dependence of π on n, the number of particles, the essence of colligative property

The process of release of neurotransmitters is highly coordinated, involving several 100 proteins with graded responses to intracellular calcium fluctuations (Goyal and Chaudhury, 2013; Südhof, 2013). In neurosecretory processes like stimulation-evoked neurotransmission, the pool of readily releasable vesicles empty contents after docking at the cell membrane of the active zones. Electron micrographs of nerve terminal varicosities always demonstrate intact membranes of vesicles within the cytosol of the terminal (Collman et al., 2015). This structural integrity of vesicles strongly suggests that the contents of the vesicles are isotonic with the matrix of the varicosities.

Earlier, an interesting study investigated the osmotic pressure of synaptic vesicles (Kopell and Westhead, 1982). This study revealed that the vesicles obtained from chromaffin cells of the adrenal gland, despite their high concentrations of various amines, and peptides, remained isotonic (Kopell and Westhead, 1982). This study hypothesized that the highly negatively charged ATP molecules, a major co-constituent of chromaffin cells beside the positively-charged amines, forms a polymeric complex within the vesicles (Kopell and Westhead, 1982).

The leading hypothesis by Estévez-Herrera et al. (2016) is that when ATP agglomerates the vesicular contents, there is a reduction in the number of free particles, leading to balance of pressures across the vesicular membrane. This physical property may be potentially a critical determinant of membrane integrity of vesicles from their biogenesis until the time they receive the necessary stimuli for exocytosis.

## SLC17A9 transports ATP into vesicles

Estévez-Herrera et al. (2016) show that SLC17A9, the vesicular nucleotide transporter (VNUT; Sawada et al., 2008), performs a rate-limiting step to the transport of ATP within the large dense core (LDC) particles of the chromaffin cells. It has been specifically demonstrated earlier and well-known for some time that the drive for ATP entry is regulated by a proton motive force (Sawada et al., 2008). Thus, it is imperative that the colligative property has a direct relationship with the intravesicular acidity.

## Enteric inhibitory smooth muscle neurotransmission involves release of vesicular ATP and *de novo* synthesized nitric oxide (NO)

**Figure 1:**
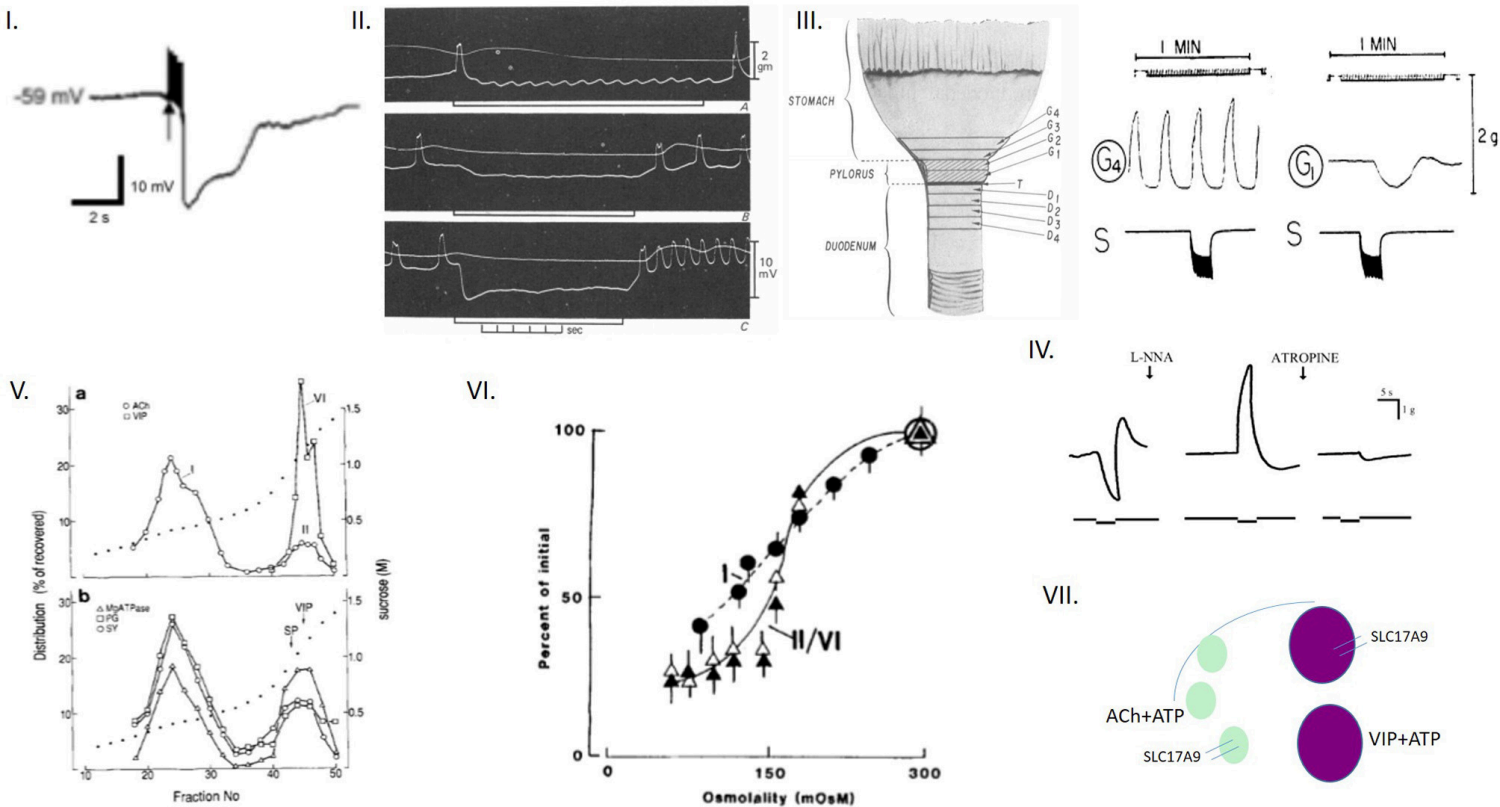
**Colligative property of ATP may have important implications for enteric inhibitory neuromuscular neurotransmission. (I)** Trace of a compound inhibitory junction potential Note the fast phase of the hyperpolarization (fast IJP, ATP mediated), followed by the slow delayed phase to repolarization (slow IJP, NO mediated). ATP is released from vesicles, whereas NO is synthesized *de novo* by nNOS. However, the identity of the ATP containing vesicles is not discretely described for myenteric axons and nerve terminals. **(II)** Three traces of electrical recordings showing differential responses to electrical field stimulation (EFS) intensity The upper trace is the mechanical recording, whereas the lower trace depicts the electrical activity. The three traces corresponds to 1, 10, and 30 Hz of stimuli, respectively. Note that at the beginning of the stimulus, inhibitory neurotransmission is observed, with hyperpolarization of the membrane potential (inhibitory junction potential, IJP). The tendency to recover to the baseline membrane potential is less with higher intensities of stimuli. The rapid phase of IJP is due to ATP. The slow phase is due to sustained synthesis of NO. However, the identity of the vesicles that releases ATP is not known. A notable feature of this recording is the excitatory junction potential (EJP) at the end of the IJP. EJPs are mainly mediated by acetylcholine. It is possible that Ach is released with the decay of the stimulus. It is also possible that Ach is released initially, but the overwhelming amount of ATP, through its postjunctional effects on the P2Y1 receptor, mediates an inhibitory response. Evidence also exists that the sustained phase of the IJP may be due to a prejunctional modulation by VIP, which is also coreleased with ATP. **(III)** Further examples of sequential relaxation and contraction during mechanical recordings G1 represents a pyloric strip, whereas G4 represents an antral strip. Note the spontaneous contractions of the antrum. In contrast, the EFS induces relaxation of the pyloric strip, which likely contributes to pyloric patency and gastric emptying in the organ *in vivo*. **(IV)** Mechanical relaxations are sensitive to L-NNA, and contractions to atropine Mechanical recordings from lower esophageal sphincter. Again, note the sequential off-contraction following an on-relaxation during the EFS (left panel). The middle panel shows an on-contraction. Combined L-NNA-atropine still manifests residual relaxation. **(V)** Enteric synaptosomal preparations show distinct vesicular compositions of acetylcholine and VIP Note that the fraction I is composed of only Ach, whereas the fraction II is composed of both Ach and VIP. The significance of this complex composition is not clear, but may potentially contribute to the excitation seen at the tail phase of an IJP. Also note that both fractions associate with Mg^2+^-ATPase, which is myosin. This could be both myosin Va and myosin II. **(VI)** Osmotic fragility of enteric synaptosomal vesicles Note that the Ach-VIP containing vesicles are slightly more fragile (as tested by incubation in a hypotonic solution) in comparison to only acetylcholine-containing vesicles, probably due to their large size. Per the recent study of Estévez-Herrera et al. (2016), ATP may importantly contribute to the osmotic stability of these vesicles. **(VII)** Cartoon depicting the potential contribution of colligative property of ATP to enteric neurotransmission This is a simplified version of what may actually exist in the enteric synaptosomes. The arc represents the active zone of the junctional membrane of the enteric varicosities. Pure ATP containing vesicles have never been detected in myenteric preparations. They either coexist with Ach, VIP or both Ach and VIP (this third kind not shown in the cartoon). ATP, via its colligative property, may contribute to the regulation of release kinetics of either Ach or VIP or both, depending upon the stimulus intensity. Reproduced with permission from Chaudhury et al. (2011); Agoston and Whittaker (1989); Anuras et al. (1974); González et al. (2004); Burnstock (1981).

The biophysical characteristics of particle-based actions of ATP may have important implications for enteric neuromuscular transmission. Evoked enteric inhibitory neuromuscular neurotransmission involves the sequential release of purines (most importantly, ATP) and the gas nitric oxide (NO), synthesized by neuronal nitric oxide synthase (nNOS) at the membranes of nerve terminals (Chaudhury et al., 2011, 2012; Chaudhury, 2014, 2015a, 2016a,b). While ATP is stored in the vesicles of the nerve terminals, NO is synthesized *de novo* (Chaudhury, 2016a). The released ATP during evoked neurotransmission hyperpolarizes the smooth muscle membrane (Chaudhury, 2016a). In a span of a few 100 ms, the membrane potential endeavors to swing back to its resting stage. However, the prolonged release of nitric oxide prevents the restoration of membrane potential to baseline and aims to maintain the hyperpolarization. This is manifested as the slow inhibitory junction potential (sIJP), unambiguously recorded by several investigators across decades (Figure 1; Bennett et al., 1966; Atanasova et al., 1972; Smith et al., 1990; Hirst et al., 2004; Allego et al., 2008; Chaudhury et al., 2011, 2012; Chaudhury, 2016a). Following the paradigm-shifting demonstration of ATP as a neurotransmitter using gut tissues (Burnstock et al., 1970), there was a gap of several decades in which the VNUT could not be identified within the synaptic vesicles. Quinacrine, the antimalarial drug, robustly stains ATP containing nerve terminals (Belai and Burnstock, 1994), but this never could provide insights into how ATP, a highly negatively charged molecule, could be shuttled across the cell membrane of the vesicles. Following the report by Sawada et al. (2008) of the molecular identity of VNUT as the solute carrier protein SLC17A9 (Sawada et al., 2008), a commercially available antibody was used to demonstrate the existence of SLC17A9 in the enteric musculomotor nerve terminals (Chaudhury et al., 2012), providing the preliminary critical evidence of fulfillment of the Sherringtonian criterion (Levine, 2007) for the existence of the transporter of a neurotransmitter.

## Importance of vesicular content clustering by ATP in diverse enteric synaptosomal vesicles

ATP is widely distributed in enteric musculomotor nerve terminals. It is present in both VIP containing large dense core vesicles, as well as acetylcholine (Ach) containing small clear vesicles (Figure 1). While VIP plays a significant role in inhibitory neurotransmission and smooth muscle relaxation, Ach facilitates excitatory neurotransmission and smooth muscle contraction. Alternate relaxation and contractions of smooth muscles at the same location are the key factors that determine transit of luminal contents but very little is known regarding the release kinetics of VIP and Ach, and parallel release of ATP. Below, we discuss some of the possibilities that may happen to execute these complex release of excitatory and inhibitory neurotransmitters during enteric nerve-smooth muscle neurotransmission. We also discuss the potential role of colligative property of ATP in influencing these functions.

- **VIP containing large dense core vesicles**: In the enteric nerve terminals, what potential colligative role does ATP play? The enteric inhibitory neurotransmission is represented electrophysiologically by the fast and slow IJP, mediated by the purine nucleotide ATP and NO, respectively (Chaudhury et al., 2011, 2012; Chaudhury, 2016a). While ATP is released from vesicles (Chaudhury et al., 2012), NO is synthesized by nNOS at the nerve terminal membrane (Chaudhury et al., 2009, 2011; Chaudhury, 2014). It is only scantily known whether other chemicals are co-released with ATP. Classical studies by Whittaker using enteric synaptosomes has demonstrated that many neuropeptides coexist with ATP. One of them is vasoactive intestinal polypeptide (VIP; Agoston et al., 1988; Whittaker, 1989). It is possible that peptide VIP is coreleased with ATP during evoked neurotransmission (Agoston and Whittaker, 1989). Though VIP may not have a direct impact on the slow IJP, studies have shown the important role of VIP in modulation of presynaptic calcium concentrations, thus having an effect on both exocytosis of ATP, as well as *de novo* synthesis of NO (Van Geldre and Lefebvre, 2004). This may be a reason for earlier erroneous suggestions of VIP as the enteric inhibitory neurotransmitter (Goyal et al., 1980; Mackenzie and Burnstock, 1980). It is possible that ATP may importantly contribute to the osmotic pressure of the VIP containing large dense core vesicles, which are similar to the chromaffin granules. This remains to be tested.
- **Acetylcholine (Ach) containing small clear vesicles (SCV):** An important aspect of coexistence of ATP in the enteric nerve terminals is that with acetylcholine (Ach). ATP, being negatively charged, can associate with the positively charged quaternary ammonium of acetylcholine. Ach contributes to excitatory junction potentials (EJPs) and contractile motor responses (Anuras et al., 1974). Intriguingly, cholinergic vesicles coexist with VIP containing large dense core vesicles (Agoston et al., 1988). The significance of this important observation is unknown. The dynamics of release of ATP/NO and Ach is also not known (Chaudhury, 2016a). A common observation is the occurrence of a contractile response at the end of an episode of relaxation during post-stimulus mechanical recordings of gastrointestinal muscle strips (Figure 1; Anuras et al., 1974). Per the previous observation, it shall imply that a given intensity of stimulus first supports inhibitory neurotransmission, followed by the cholinergic excitatory response. By the time the excitatory response appears, the initial stimulus would start decaying temporally. But what prevents simultaneous release of Ach during ATP release? It has been shown that low frequency electrical field stimulation of synaptosomes *ex vivo* released ACh by < four-fold the basal release; the simultaneously detected VIP secretion was only slightly raised above the basal level. During high frequency stimulation (50 Hz), VIP secretion was greatly increased (to five-fold the resting release) whereas the release of ACh increased to only 150% of the basal output (Agoston and Whittaker, 1989). An alternate possibility is that both ATP and Ach are coreleased, and depending on the postjunctional responses, there is an inhibitory or excitatory response. Sometimes, say during segmentation contractions, a long stretch may simultaneously have sustained inhibitory purinergic–nitrergic responses (Gwynne and Bornstein, 2007). How is cholinergic responses excluded during this activity? In the light of these perspectives, the reductionist concepts of descending inhibitory neurotransmission and ascending excitatory neurotransmission merits critical revision. How do the circuits toggle between an excitatory vs. inhibitory prejunctional release? This may also result from a summative response. Though the current concepts limit us to thinking that the postjunctional smooth muscle responses are somewhat chaotic and stochastic in nature, there is potential stoichiometry to how nature must have designed these enteric circuits, including specific responses to intraluminal stimuli, and responses mediated by intrinsic primary afferent neurons (IPANs). SLC17A9 colocalizes with vesicular acetylcholine transporter (Chaudhury et al., 2012). Again, it remains to be examined whether ATP contributes to colligative actions with the acetylcholine containing vesicles.

## Impact of colligative property of ATP on differential release of enteric excitatory and inhibitory neurotransmitters and neuromodulators

The biophysical experiments of estimating osmotic pressure of vesicles are challenging to perform, and more so in an *in vivo* context. Enteric synaptosomal preparations may be used to examine whether the mechanisms of ATP contributing to particle stability (Estévez-Herrera et al., 2016) is a general phenomenon seen across all vesicular structures, for example cholinergic containing small synaptic vesicles and VIP containing large dense core vesicles. Additionally, polymeric vesicular contents with (ATP-neurotransmitter)_n_ needs demonstration, likely by estimation of the polymeric masses or by surrogate measures of vesicular acidity. The specific gravity of the clear and dense core vesicles have been reported (Table 1). A relevant hypothesis that may be examined is whether the ATP contents are different between exclusive Ach containing vesicles vs. Ach-VIP containing vesicles. If so, the particle aggregating effects of ATP may differentially regulate release of Ach and VIP during excitatory and inhibitory neurotransmission, respectively.

**Table 1:** **Table showing the relative specific gravity of different enteric synaptic vesicles**.

**Enteric Neurotransmitter**	**Ach (acetylcholine)**	**Substance P**	**Somatostatin**	**VIP (vasoactive intestinal polypeptide)**
Mean density (g/ml)	1.066	1.123	1.138	1.148
Vesicle diameter (nm)	61	65	37	110

The recent study by Estévez-Herrera et al. (2016) suggest that ATP may contribute to the osmotic stability of these vesicles. Data obtained from Agoston et al. (1985).

## Pathophysiological implications of ATP colligative property for functional bowel disorders: lessons may be learnt from SLC17A9^−/−^ mice

Most esophagogastrointestinal motility disorders involve dysfunction of nitrergic biosynthesis and postjunctional smooth muscle responses (Chaudhury, 2015a,b, 2016b). Varied mechanisms of pathophysiology finally converge on the nitrergic pathways to cause diseases like achalasia, gastroparesis, pseudo-obstruction, megacolon, and constipation. There are virtually no disorders in which purinergic inhibitory neurotransmission has been found as the solitary basis of the gastrointestinal motility disorder. There are incipient suggestions that the purinergic fast IJP may be impaired, for example in the transitional zone in Hirschsprung's disease (Jiménez et al., 2015). It is possible that defective ATP production or vesicular shuttle may cause subtle defects in inhibitory neuro-smooth muscle neurotransmission. Mitochondrial ATP production is defective in diabetes (Bagkos et al., 2014). ATP gates SLC17A9 (Sawada et al., 2008). Thus, deficient ATP production may cause SLC17A9 channelopathy. SLC17A9 knockout mice do not show any frank gastrointestinal phenotypic abnormalities and have normal body weight (Dr. Richard Palmiter, personal communication). In SLC17A9 knockout mice, insulin vesicular exocytosis is accelerated (Sakamoto et al., 2014). This may result from deficient particle aggregating action of ATP. Whether such defects also occur in enteric vesicular release remains to be tested. We plan to undertake further studies of the enteric synaptosomal properties during neurotransmission to test the generalizability of colligative property of ATP and any effect of its deficiency on purinergic neuromuscular transmission.

## Author contributions

AC conceptualized and drafted manuscript. VD important intellectual participation. WM important intellectual participation, overall supervision. All authors read and approved final version of manuscript.

### Conflict of interest statement

The authors declare that the research was conducted in the absence of any commercial or financial relationships that could be construed as a potential conflict of interest.
